# Patterns of insecticide resistance and knock down resistance (*kdr*) in malaria vectors *An. arabiensis*, *An. coluzzii* and *An. gambiae* from sympatric areas in Senegal

**DOI:** 10.1186/s13071-016-1354-3

**Published:** 2016-02-05

**Authors:** El Hadji Amadou Niang, Lassana Konaté, Mawlouth Diallo, Ousmane Faye, Ibrahima Dia

**Affiliations:** Unité d’Entomologie Médicale, Institut Pasteur de Dakar, 36 Avenue Pasteur, BP 220, Dakar, Sénégal; Laboratoire d’Ecologie Vectorielle et Parasitaire, Université Cheikh Anta Diop de Dakar, Dakar, Sénégal

**Keywords:** Insecticide resistance, kdr resistance, *Anopheles arabiensis*, *Anopheles coluzzii*, *An. gambiae*

## Abstract

**Background:**

Malaria vector control in Africa relies on insecticides targeting adult mosquito vectors via insecticide treated nets or indoor residual spraying. Despite the proven efficacy of these strategies, the emergence and rapid rise in insecticide resistance in malaria vectors raises many concerns about their sustainability. Therefore, the monitoring of insecticide resistance is essential for resistance management strategies implementation. We investigated the *kdr* mutation frequencies in 20 sympatric sites of *An. arabiensis* Patton, *An. coluzzii* Coetzee & Wilkerson and *An. gambiae* Giles and its importance in malaria vector control by evaluating the susceptibility to insecticides in four representative sites in Senegal.

**Methods:**

Sibling species identification and *kdr* mutation detection were determined using polymerase chain reaction on mosquitoes collected using pyrethrum sprays collection in 20 sites belonging to two transects with differential insecticide selection pressure. The World Health Organization (WHO) tube test was used to determine phenotypic resistance of *An. gambiae* s.l. to DDT, deltamethrin, lambdacyholothrin, permethrin, bendiocarb and malathion in four representative sites.

**Results:**

The L1014F *kdr* mutation was widely distributed and was predominant in *An. gambiae* in comparison to *An. arabiensis* and *An. coluzzii*. The bioassay tests showed a general trend with a resistance to DDT and pyrethroids and a susceptibility to organophosphate and carbamate according to WHO thresholds. For deltamethrin and permethrin, the two most used insecticides, no significant difference were observed either between the two transects or between mortality rates suggesting no differential selection pressures on malaria vectors. The study of the KD times showed similar trends as comparable levels of resistance were observed, the effect being more pronounced for permethrin.

**Conclusions:**

Our study showed a widespread resistance of malaria vectors to DDT and pyrethroids and a widespread distribution of the 1014F *kdr* allele. These combined observations could suggest the involvement of the *kdr* mutation. The existence of other resistance mechanisms could not be ruled out as a proportion of mosquitoes did not harbour the *kdr* allele whereas the populations were fully resistant. The susceptibility to carbamate and organophosphate could be exploited as alternative for insecticide resistance management.

## Background

Malaria vector control in Africa is based predominately on the use of residual insecticides through indoor residual spray and insecticide treated nets [1]. Both methods have shown to be very effective against *Anopheles* mosquitoes [2, 3]. Pyrethroids are considered most suitable for bednets impregnation due to their insecticidal effect, relative safety for human and other mammals and their quick knock-down effect on mosquitoes, whereas other insecticide classes (organophosphates, carbamates and organochlorines) are mainly used for Indoor Residual Spraying [4]. In some contexts, the use of both methods has significantly improved the prevention and control of malaria [3]. However, the development of insecticide resistance has become a serious threat to the effectiveness of these control measures. One of the mechanisms involved in pyrethroid resistance in *Anopheles gambiae* Giles is caused by target-site insensitivity through a knock-down resistance (*kdr*) produced by two different points mutation at amino acid position 1014 of the voltage gated sodium channel gene. The first leads to leucine-to-phenylalanine substitution and is widely distributed in West Africa [5] whereas the second was described in East Africa and involves a leucine-to-serine change [6]. To date, the latter is mainly found with the 1014F *kdr* allele and is suspected to be less involved in pyrethroid resistance than the 1014F allele [7, 8]. Within the *An. gambiae* complex, a sympatric ecological diversification is in progress and has leaded to the emergence of at least two incipient species (the M and S molecular forms). These forms repeatedly showed heterogeneous levels of divergence in most parts of Africa [9, 10], and are now recognised as separate species - *An. coluzzii* Coetzee & Wilkerson and *An. gambiae* respectively for M and S forms [11]. In their “far west” African distribution, genetic isolation was observed along the Senegambian coasts [12] as well as in inland areas of south-eastern Senegal, where substantial reproductive isolation was evident and further supports the ongoing process of speciation in inland areas [13].

Previous studies have revealed the presence of the *kdr* mutation in *An. gambiae* and its absence in *An. coluzzii* populations even in sympatric areas where the mosquitoes are presumably subjected to similar selection pressures. These observations supported the possibilities of different resistance mechanism in *An. coluzzii* populations or a restriction in gene flow between the two species [14–17] that could therefore affect the spread of the *kdr* mutation. Subsequent studies conducted thereafter showed the presence of this mutation in *An. coluzzii* populations in several sites in Africa. This was first reported in Benin and was attributed to the existence of gene flow from *An. gambiae* to *An. coluzzii* rather than the same mutation that occurred in both species [18] and further in Cameroon [19].

Based on the presence of *An. arabiensis* Patton, *An. coluzzii* and *An. gambiae* in sympatric areas in south-eastern Senegal, we assessed the *kdr* frequencies in 20 sites and further studied its importance in terms of vector control by evaluating the susceptibility to insecticides in four representative sites.

## Methods

### Study design and study sites

**Fig. 1:**
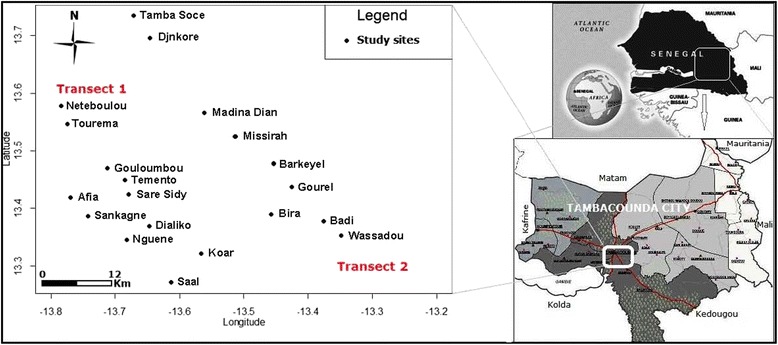
Localisation of the study sites

The study was conducted in the Tambacounda region of southeastern Senegal (Fig. 1) during the rainy seasons, 2010 and 2011, which last from June to October, with a peak in August–September, and an annual rainfall of 500 mm.

**Fig. 2:**
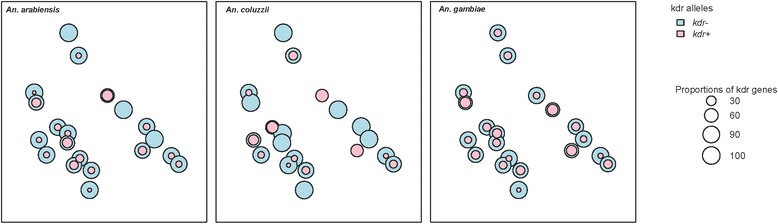
Spatial variations of the L1014F *kdr* allele in *An. arabiensis*, *An. coluzzii* and *An. gambiae* in the 20 sites prospected

In 2010, 20 sites were selected from two distinct transects. The first is situated along the Gambia River an agricultural areas growing rice and cash crops of banana with high pesticide usage. The second transect is along the National Road 7, in a less humid and mostly arid area with a little or no application of pesticides. In each site, indoor resting mosquitoes were collected during the daytime using pyrethrum sprays. Upon collection, mosquitoes were counted and identified as *An. gambiae* s.l. morphologically using the keys of Gillies & de Meillon [20]. All the mosquito samples were stored individually after identification in numbered vials containing desiccant until laboratory processing.

In 2011, *Anopheles* larvae and pupae were collected from natural breeding sites in four selected sites (two in the first transect namely Koar and Sankagne and two in the second transect namely Djnkore and Wassadou). Upon collection, they were kept in separate labelled bottles, transported to the insectary and maintained at a relative humidity of 75 ± 5 % and a temperature of 28 ± 3 °C.

### *kdr* molecular genotyping and species identification

For molecular identification, genomic DNA was extracted from the wings or legs of individual mosquitoes as described by Collins et al. [21]. For each collection of mosquitoes randomly sampled from each village, 30–100 % of females belonging to the *An. gambiae* s.l. were identified to species using the molecular methods of Favia et al. [22] and Fanello et al. [23]. The presence of L1014F mutation was confirmed using the method described by Martinez-Torres et al. [5].

### Insecticide susceptibility tests

Insecticide susceptibility tests were conducted on unfed adult females aged from 2 to 5 days. Bioassays were carried out using WHO test kits for adult mosquitoes. Insecticide-impregnated papers were provided by The Vector Control Research Unit, School of Biological Sciences (Universiti Sains Malaysia), a WHO Collaborating Centre. The following diagnostic concentrations of insecticides were tested: 0.05 % deltamethrin, 4 % DDT, 0.75 % permethrin, 0.05 % lambdacyhalothrin, 5 % malathion and 0.1 % bendiocarb. For each insecticide, four replicates were exposed for 60 min. The mosquitoes were then transferred in tubes with untreated papers and kept under observation for 24 h.

### Data analysis

The *kdr* allele frequency was estimated for each site and period of collection as the proportion of specimens found with the L1014F *kdr* alleles. The frequencies as well as the conformity to Hardy–Weinberg equilibrium were assessed using Genepop v.3.2. [24].

For each insecticide, the resistance status was studied using WHO criteria [1]. For DDT and pyrethroid insecticides, knock-down rates (%) were assessed at 10, 15, 20, 30, 40, 50 and 60 min.

All statistical analyses were performed using R software (version 3.0.2).

## Results

### Frequency and distribution of 1014F allele

In this study, the *kdr* mutation was confirmed in *An. gambiae* s.l. populations in all of the 20 sites selected (frequency range 0.05–0.33 %) with significant variations (χ^2^ = 76.3, df = 19, *p* < 0.001). Examination of 189 *An. arabiensis*, 115 *An. coluzzii* and 814 *An. gambiae* showed that only *An. gambiae* s.s. was carrying the *kdr* mutation in all the 20 sites (frequency range: 0.06–0.38 %). The *kdr* mutation was observed in 17 sites (frequency range in observed sites: 0.04–0.40 %) and 12 sites (frequency range in observed sites: 0.06–0.50 %) in *An. arabiensis* and *An. coluzzii,* respectively (Table 1, Fig. 2). The mean *kdr* frequencies by species were 14.38 ± 2.42 (*An. arabiensis*), 21.22 ± 3.85 (*An. gambiae*) and 14.69 ± 1.95 (*An. coluzzii*). No significant differences were observed between these means (one-way ANOVA, F = 1.83, *p* = 0.17).

**Table 1:** Frequencies of the L1014F *kdr* allele within the 20 sites prospected

Transects	Sites	*An. arabiensis*				*An. gambiae*				*An. coluzzii*			
		n	Fis	Freq	95 % CI	n	Fis	Freq	95 % CI	n	Fis	Freq	95 % CI
Transect 1	Neteboulou	23	−0.05	0.04	0.01–0.15	30	−0.25	0.20	0.11–0.32	4	−0.14	0.13	0.00–0.53
	Tourema	13	**0.80**	0.27	0.12–0.48	21	**0.80**	0.38	0.24–0.54	1	0	0.00	0.00–0.54
	Gouloumbou	57	**0.65**	0.15	0.09–0.23	76	**0.50**	0.22	0.15–0.29	13	**0.84**	0.42	0.23–0.63
	Afia	32	**0.63**	0.09	0.04–0.19	35	0.03	0.16	0.08–0.26	3	−0.5	0.33	0.04–0.78
	Temento	13	−0.13	0.12	0.02–0.30	27	−0.2	0.28	0.16–0.42	0	0	0.00	-
	Sare Sidy	20	0.43	0.33	0.19–0.49	38	**0.42**	0.20	0.11–0.30	1	0	0.00	0.00–0.84
	Sankagne	21	0.32	0.12	0.04–0.26	23	0.40	0.24	0.13–0.39	7	**1**	0.14	0.02–0.43
	Nguene	29	**0.58**	0.21	0.11–0.33	40	0.12	0.11	0.05–0.20	8	**1**	0.13	0.02–0.38
	Dialiko	19	**0.59**	0.24	0.11–0.40	45	0.17	0.20	0.12–0.30	9	−0.06	0.06	0.00–0.27
	Koar	44	**0.37**	0.15	0.08–0.24	103	**0.38**	0.25	0.19–0.32	20	**0.69**	0.20	0.09–0.36
	Saal	10	−0.05	0.05	0.00–0.25	34	**0.47**	0.06	0.02–0.14	8	0	0.00	0.00–0.21
Transect 2	Tamba Soce	19	-	0.00	0.00–0.09	14	**0.76**	0.18	0.06–0.37	0	0	0.00	-
	Djnkore	42	**0.45**	0.10	0.04–0.18	65	**0.61**	0.20	0.14–0.28	2	−0.33	0.25	0.01–0.81
	Madina Dian	20	**0.79**	0.4	0.25–0.57	17	**0.76**	0.15	0.05–0.31	2	1	0.50	0.07–0.93
	Missirah	5	-	0.00	0.00–0.31	4	0.47	0.38	0.09–0.76	0	0	0.00	-
	Barkeyel	27	**1**	0.15	0.07–0.27	22	**0.74**	0.23	0.11–0.38	2	0	0.00	-
	Gourel	6	-	0.00	0.00–0.26	59	0.11	0.14	0.09–0.22	4	0	0.00	-
	Bira	13	0.13	0.23	0.09–0.44	30	0.05	0.35	0.23–0.48	2	0	0.50	0.07–0.93
	Badi	41	**0.54**	0.12	0.06–0.21	56	0.12	0.09	0.04–0.16	24	**0.45**	0.08	0.02–0.20
	Wassadou	16	**0.63**	0.13	0.04–0.29	75	−0.02	0.24	0.17–0.32	5	−0.25	0.20	0.03–0.56

*n* number of specimens, F_is_ : inbreeding coefficient calculated according to Weir and Cockerham [40], F_is_ < 0 indicate an excess of heterozygotes, F_is_ > 0 denote heterozygotes deficiency, values in bold indicate significant deviation from Hardy–Weinberg (*P* < 0.05)

These frequencies were significantly different only between *An. gambiae* and *An. arabiensis* (χ^2^ = 10.92, df = 1, *p* = 0.0009), irrespective of the transects. When considering each species within each different transect, the prevalence of the 1014F *kdr* allele was similar between all three species in each transect. In *An. coluzzii*, the 1014F *kdr* allele was the only allele observed in two of the nine localities prospected in transect two (Madina Dian and Bira) .

### Mortality rates

In all the bioassays, controls using wild *An. gambiae* s.l. populations from each site showed mortality rate less than 5 %, thus, no corrections were required in the test sample data.

**Fig. 3:**
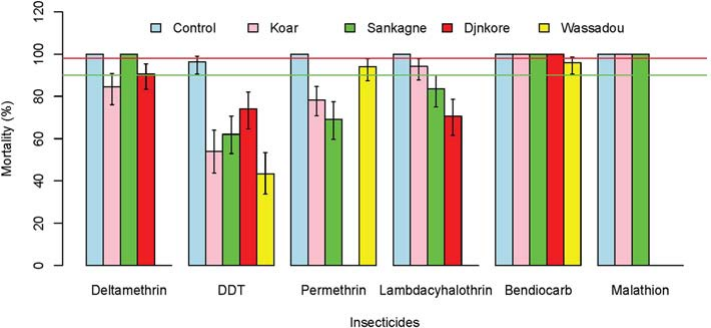
Mortalities observed with the six insecticides tested for the four populations. The *green and red lines* indicate respectively the 90 and 98 % limits

All populations exhibited resistance to DDT (Fig. 3). The highest mortality rate was observed for Djnkore populations (mortality rate = 74.07 %, IC95 = 64.75–82.03 %). For pyrethroids, resistance was observed for both types: Type I (permethrin) and Type II (deltamethrin, lambdacyhalothrin) for all populations except in Sankagne (deltamethrin) and probably in Wassadou (permethrin) and Koar (lambdacyhalothrin) where a probable resistance is observed with the mortality rates of 94 % (IC95 = 87.4–97.77 %) and 94.12 % (IC95 = 87.64–97.81 %) for the two latter villages. Full susceptibility was observed for the organophosphate malathion (Koar and Sankagne) and for the carbamate bendiocarb (Koar, Sankagne and Djnkore). Wassadou populations exhibited resistance to bendiocarb with a mortality rate of 95.87 % (IC95 = 90.62–98.64 %).

Although a statistically significant difference was observed between mortality rates for sites showing confirmed resistance for DDT (χ2 = 22.3, df = 3, *p* < 0.001), no significant differences were observed for deltamethrin and permethrin, whereas mortality rates to lambdacyhalothrin were significantly different between Sankagne and Djnkore (χ2 = 4.4, df = 1, *p* = 0.03).

### Knock down effects of pyrethroids

**Fig. 4:**
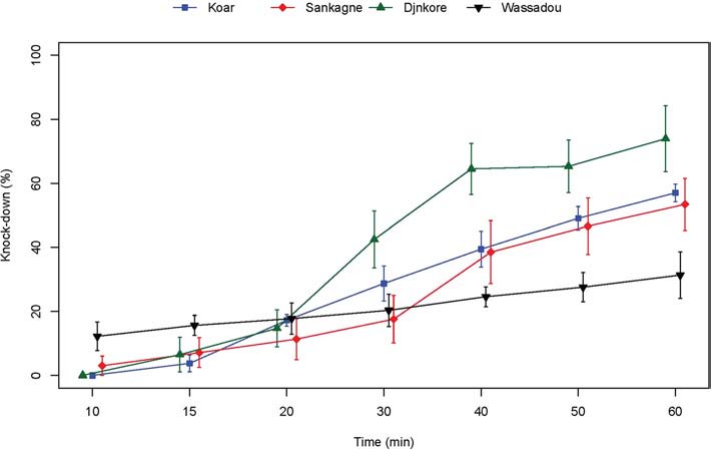
Evolution of the knock-down rates of mosquitoes due to exposure to DDT

**Fig. 5:**
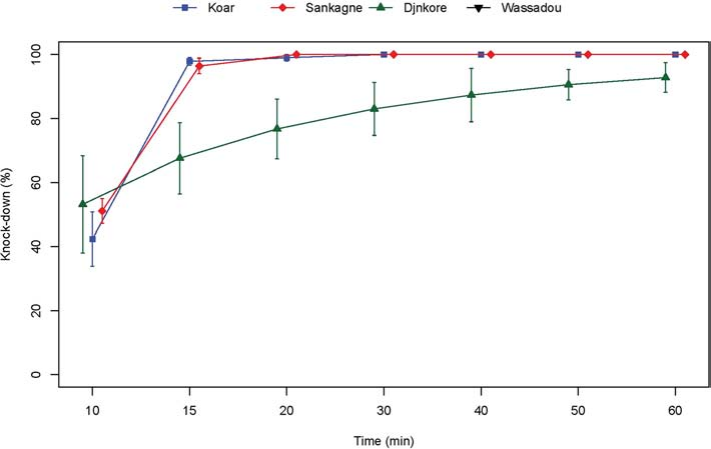
Evolution of the knock-down rates of mosquitoes due to exposure to deltamethrin

**Fig. 6:**
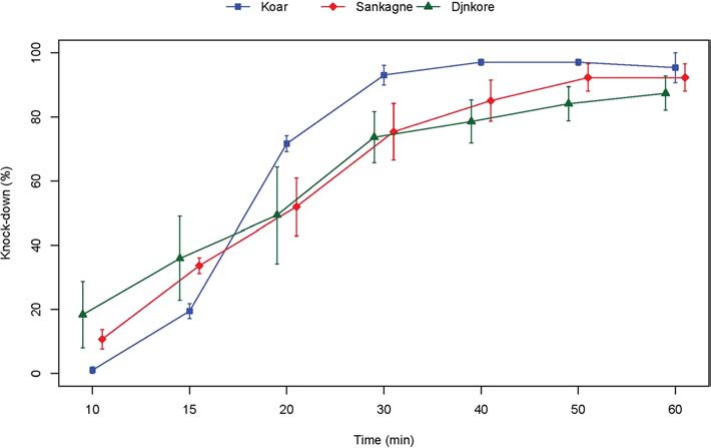
Evolution of the knock-down rates of mosquitoes due to exposure to lambdacyhalothrin

**Fig. 7:**
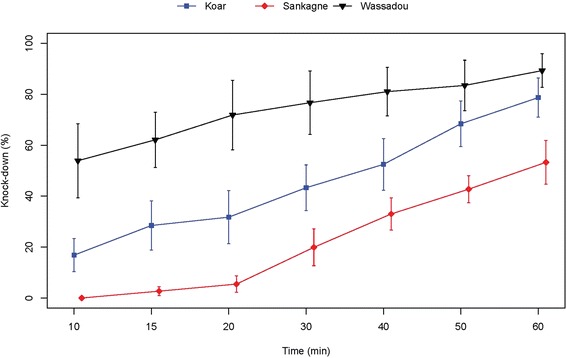
Evolution of the knock-down rates of mosquitoes due to exposure to permethrin

Despite the resistant status observed, the study of the knock down effects of DDT and pyrethroids showed different patterns. For DDT, the resistance observed was associated to a low effect on the four populations tested (Fig. 4). Indeed after 60 min exposure the highest knock down effect was below 75 % (population from Djnkore). For the other populations, the highest values were respectively 57.01, 53.43 and 31.35 % at 60 mn for Koar, Sankagne and Wassadou (Fig. 4). For these populations, KD50 and KD95 increased markedly with a near complete loss of knock down effect for the Wassadou population (Table 2). Despite the resistance status observed for Koar, a similar trend of the KD rates was observed for deltamethrin in Sankagne (*p* < 0.05) with 100 % knock down at 20 min (Fig. 5). For lambdacyhalothrin, the KD dynamics were quite similar for Koar, Sankagne and Djnkore populations (Fig. 6). While the KD50 times were similar, a slight increase of the KD95 was observed in the resistant population of Djnkore, but no significant difference was observed with the populations of Koar and Sankagne (*p* < 0.05 after Bonferroni correction). For permethrin, the pattern of KD effect is similar to the resistance status (Fig. 7). Whatever the population pairs considered, the difference was statistically significant (*p* < 0.05).

**Table 2:** Observed knock-down for DDT and the pyrethroids tested

Insecticides		Djnkore	Koar	Sankagne	Wassadou	Control strain
DDT	Number tested	108	100	124	106	106
	KD50 (min)	34.57	49.52	52.37	no kd	20.06
		(31.05–38.71)	(44.93–55.51)	(43.94–68.50)		(18.25–21.98)
	KD95 (min)	90.54	174.86	197.56	no kd	35.13
		(73.57–123.55)	(136.28–247.58)	(126.78–458.52)		(30.81–42.71)
deltamethrin	Number tested	106	103	107		103
	KD50 (min)	7.79	10.28	9.93	-	10.93
		(1.59–12.69)	(9.71–10.77)	(9.31–10.43)		(10.26–11.53)
	KD95 (min)	73.27	14.71	14.43	-	17.25
		(43.77–403.87)	(13.72–16.34)	(13.45–16.12)		(16.00–19.17)
lambdacyhalothrin	Number tested	119	102	103		101
	KD50 (min)	21.42	19.12	20.19	-	16.63
		(16.39–26.46)	(17.14–21.16)	(18.01–22.37)		(15.90–17.42)
	KD95 (min)	85.91	38.41	55.10	-	20.20
		(59.22–181.26)	(33.16–47.55)	(46.79–69.03)		(19.00–22.47)
permethrin	Number tested	-	147	113	100	102
	KD50 (min)	-	31.89	55.61	7.35	9.05
			(25.28–41.89)	(49.46–65.29)	(0.09–14.00)	-
	KD95 (min)	-	194.85	164.88	138.15	10.33
			(109.60–698.53)	(123.03–262.62)	(59.42–76.10^3^)	-

## Discussion

The present study compared the patterns of insecticide resistance and *kdr* resistance in three sympatric species of the *An. gambiae* complex. The study of the distribution of the L1014F *kdr* mutation showed its widespread presence in the study sites particularly in *An. gambiae* in comparison to *An. arabiensis* and *An. coluzzii* as observed elsewhere in Africa [7, 8, 25]. In Senegal, previous reports have documented the presence of the L1014F *kdr* allele in *An. gambiae* populations from the southeastern part of the country with frequencies ranging from 14.1 to 18.6 % [26] and in *An. arabiensis* populations from two suburbs of Dakar [27]. Compared to the present study, these observed frequencies are low with respect to some of our sites herein, where the L1014F *kdr* mutation frequencies were up to 38 % in *An. gambiae*, 50 % in *An. coluzzii* and 40 % in *An. arabiensis*. However, these frequencies seem to be low when compared with other sites in Benin where frequencies of up to 80 % were recently observed [28]. The comparison of the observed frequencies with those expected under Hardy-Weinberg equilibrium indicated in some cases (mainly in *An. gambiae* and *An. arabiensis* populations), a deficit or excess of heterozygotes. While the origin of the L1014F mutation in *An. arabiensis* is suspected to be a new and independent mutation, its presence in *An. coluzzii* has been suggested to occur by introgression from *An. gambiae* [29]. Therefore, it is undoubtedly important to carry out additional studies to unravel the origin of this mutation in *An. arabiensis* and *An. coluzzii* in our context as other mechanisms could be involved.

Globally, the results of the bioassay tests showed that the populations were resistant to DDT and pyrethroids and susceptible to organophosphate and carbamate. This situation is relatively common in many sites in Africa [30]. In West Africa, pyrethroid resistance is high, widespread and predominant in *An. gambiae* compared to *An. arabiensis* [8]. Concerning carbamate and organophospahe insecticides, our results contrast with recent findings that showed high levels of resistance to these insecticides in the urban site of Dakar [31].

The resistance levels observed to pyrethroids varied greatly between the four sites studied. For deltamethrin and permethrin the lack of difference in mortality rates could reflect no differential selection pressures on malaria vectors. This could be due to the fact that these insecticides are the most widely used in insecticides-treated nets distributed by the National Malaria Control Program in Senegal. Indeed, Senegal is on the short list of African countries that have reached the RBM target of 80 % of households owning at least one insecticide-treated nets [32]. Despite the resistance observed, the study of the KD dynamic showed a similar effect of deltamethrin on the resistant population of Koar compared to the susceptible population of Sankagne where a similar trend was observed. In contrast, the study of the effect of permethrin showed profiles similar to observed resistance. Except the fact that deltamethrin and permethrin are different chemical molecules (type I vs type II), this observation may express a more recent use of deltamethrin in the area in comparison with permethrin. Indeed, impregnated-bednets (Olyset Net©) were introduced in the study area in the 1990’s [33] and could have exerted strong insecticidal pressures on mosquitoes giving rise to the high levels of insecticide resistance observed. However, for lambdacyhalothrin, the difference observed between Koar and Djnkore reflects variation in resistance selection pressures, which results from differential use in agriculture representing the main human activities.

## Conclusions

During this study a widespread resistance to DDT and pyrethroids and a widespread 1014F *kdr* allele distribution was observed. The observed resistance to DDT and pyrethroids combined with the high KD times could suggest the involvement of the 1014F *kdr* mutation, as observed elsewhere [34–37]. However, the involvement of other resistance mechanisms including the 1014S *kdr* mutation recently observed in West Africa and Senegal [31, 38, 39], could not be ruled out, as at least 50 % of the populations did not harbour the 1014F allele whereas the populations were fully resistant. This needs further investigations. The susceptibility to carbamate and organophosphate could be an alternative for insecticide resistance management.
